# Ultrasound Thawing Optimization as a Novel Strategy to Improve Quality of Slowly Frozen Chicken Breast

**DOI:** 10.3390/foods14193446

**Published:** 2025-10-08

**Authors:** Suelen Priscila Santos, Silvino Sasso Robalo, Monica Voss, Bianca Campos Casarin, Bibiana Alves dos Santos, Renius de Oliveira Mello, Juliano Smanioto Barin, Cristiano Ragagnin de Menezes, Paulo Cezar Bastianello Campagnol, Alexandre José Cichoski

**Affiliations:** 1 Department of Food Science and Technology, Federal University of Santa Maria, Av. Roraima Nº1000, Santa Maria 97105-900, RS, Brazil; suelenp_@hotmail.com (S.P.S.); silvinosr@gmail.com (S.S.R.); bianca.casarin@acad.ufsm.br (B.C.C.); bialvesantos@gmail.com (B.A.d.S.); renius.mello@ufsm.br (R.d.O.M.); juliano@ufsm.br (J.S.B.); cristiano.ufsm@gmail.com (C.R.d.M.); paulo.campagnol@ufsm.br (P.C.B.C.); 2 Department of Animal Science, Federal University of Santa Maria, Av. Roraima Nº1000, Santa Maria 97105-900, RS, Brazil; 3 Federal Institute of Education, Science, and Technology of Roraima, Av. Glaycon de Paiva, 2496—Pricumã, Boa Vista 69303-340, RR, Brazil; monicavoss-tca@hotmail.com

**Keywords:** ultrasound, slow freezing, thawing, quality, color, drip loss

## Abstract

Chicken meat is highly consumed worldwide due to its nutritional value, but its high water content and abundance of polyunsaturated fatty acids make it particularly vulnerable to structural and oxidative damage during freezing and thawing. Slow freezing, in particular, generates large ice crystals that severely impair water-holding capacity (WHC), increase drip loss, promote color deterioration, and intensify protein and lipid oxidation. Innovative thawing strategies are therefore required to mitigate these quality losses. Ultrasound (US) has been successfully applied to accelerate thawing of fast-frozen meat; however, its potential for slowly frozen chicken breast remains poorly understood. This study aimed to evaluate the effects of US-assisted thawing at two frequencies (25 and 130 kHz), two amplitudes (100% and 60%), and three operating modes (normal, sweep, and degas) on the quality of slowly frozen chicken breast. Conventional thawing required 50 min, yielding WHC of 9.87%, drip loss of 4.65%, free sulfhydryls of 16.38 µmol/g, and ∆E of 3.91. In contrast, the optimized US condition (25 kHz, 100% amplitude, sweep mode) thawed samples in only 18 min, with markedly improved WHC (23.14%), reduced drip loss (3.25%), higher preservation of free sulfhydryls (24.69 µmol/g), and minimal color change (∆E = 3.72). Conversely, less effective parameters (e.g., 130 kHz, 60% amplitude, normal mode) prolonged thawing and compromised quality, with WHC dropping to 9.96% and drip loss increasing to 9.05%. Overall, US reduced thawing time under all conditions, but quality responses depended strongly on the applied parameters. The present findings demonstrate the novelty of optimizing US frequency, amplitude, and mode for thawing slowly frozen chicken breast, highlighting sweep mode at 25 kHz and 100% amplitude as the most effective strategy. Future research should explore its scalability and industrial applicability for poultry processing.

## 1. Introduction

Chicken meat represents one of the most widely produced and consumed animal proteins worldwide, with global production exceeding 120 million metric tons annually and consumption continuing to rise due to its affordability and accessibility. Nutritionally, chicken is valued for its high-quality proteins, essential amino acids, vitamins (such as B-complex), and minerals, while being relatively low in fat compared to red meats. However, its high proportion of polyunsaturated fatty acids and moisture content (60–70%) also make it more susceptible to lipid and protein oxidation, microbial spoilage, and textural deterioration [1,2]. To ensure product safety and maintain quality, preservation strategies are essential in poultry processing. Among them, freezing is the most effective method, as it slows down microbial growth and biochemical reactions. Nevertheless, slow freezing promotes the formation of large ice crystals, causing structural damage to muscle fibers, leading to increased drip loss, reduced water-holding capacity, and color changes. Therefore, the subsequent thawing stage becomes a critical step: depending on the thawing method, quality losses may be minimized or exacerbated.

Rapid freezing generates small ice crystals that cause only minor alterations. In contrast, slow freezing and temperature fluctuations during frozen storage favor recrystallization into large ice macrocrystals, leading to cellular disruption, water release, and subsequent quality loss [3,4]. These structural damages exacerbate lipid and protein oxidation, particularly during thawing, when ice melting and oxygen exposure promote the formation of reactive oxygen species (ROS) such as ·OH and ROO· radicals [5]. Protein oxidation may also initiate or be initiated by lipid oxidation, with aldehydes and hydroperoxides interacting with amino acids to generate oxidatively modified proteins [6].

Chicken breast muscle is especially prone to oxidative stress due to its mixed fiber composition and the high presence of oxidative fibers with ferryl myoglobin radicals, which catalyze lipid and protein oxidation [7,8]. Consequently, the loss of water-holding capacity (WHC), increased drip loss, color deterioration, and reduced juiciness are frequent outcomes in slowly frozen and thawed meat.

Conventional thawing methods, such as air thawing, immersion in water, slow refrigeration, or microwave heating, are widely applied in the food industry. However, these techniques are often associated with undesirable effects, including moisture loss, protein denaturation, lipid and protein oxidation, and, in some cases, microbial proliferation [9]. To mitigate such drawbacks, innovative thawing methods have been explored, with ultrasound (US) emerging as a promising alternative. Ultrasound refers to sound waves with frequencies above 20 kHz, typically applied in the 20–50 kHz range for food processing. Its mechanism is based on acoustic cavitation: the rapid formation, growth, and collapse of microbubbles in the medium generate localized high shear forces, turbulence, and microjets. These physical effects enhance heat and mass transfer, leading to more uniform thawing and significantly reduced thawing times [10]. Furthermore, ultrasound can preserve water-holding capacity and limit oxidative reactions, thereby contributing to improved quality retention [11]. Nevertheless, the outcomes are strongly dependent on processing parameters. Moderate ultrasound amplitudes enhance thawing efficiency and quality, whereas excessive intensity or prolonged exposure may cause localized overheating, free radical generation, and structural damage to proteins and lipids [12,13,14]. Thus, careful optimization of amplitude, frequency, and treatment time is critical to fully exploit the benefits of ultrasound while minimizing its potential adverse effects.

Most previous studies on ultrasound-assisted thawing have focused on rapidly frozen meat, where ultrasound has been shown to effectively accelerate thawing and preserve quality [15,16]. However, its efficacy in slowly frozen chicken breast remains poorly understood, despite the fact that slow freezing is commonly encountered in both domestic and industrial contexts and often leads to severe quality deterioration due to the formation of abundant intra- and extracellular macrocrystals [17]. Therefore, the present study aimed to systematically evaluate the effects of ultrasound-assisted thawing under different processing conditions, including two frequencies (25 and 130 kHz), two amplitudes (100% and 60%), and three bath operating modes (normal, sweep, and degas). The parameters analyzed comprised thawing time, drip loss, moisture content, water-holding capacity (WHC), pH, redox potential (Eh), texture, myoglobin redox states, instrumental color, and protein oxidation. By addressing this gap, the study provides novel insights into the optimization of ultrasound-assisted thawing and highlights its potential as a practical strategy to improve poultry processing.

## 2. Materials and Methods

### 2.1. Sample Preparation

Chicken breasts (m. pectoralis major) were obtained from a local slaughterhouse 24 h post-mortem, a period corresponding to the post-rigor condition (pH < 6.0), and were transported in a thermal bag with dry ice at 4 °C to the laboratory. Cylindrical portions (5 cm diameter × 1.5 cm thickness; average weight 36.0 ± 1.0 g) were obtained from the medial region of the muscle and individually packed in plastic bags (10 cm width × 10 cm length) composed of polyethylene (PE) and nylon (PA) in the sequence PE/PA/PE, with a thickness of 100 µm. The films had an oxygen permeability rate (TPO2 < 50 cc/m^2^/day at 23 °C) and a water vapor permeability rate (TPVA ≤ 10 g/m^2^/day). Packages were vacuum-sealed. Samples were subjected to slow freezing at −20 °C in a freezer (Electrolux^®^, model FE26, São Paulo, Brazil) for 10 days. In each cylindrical chicken breast sample, thermocouples (Minipa MTK-15B, type K, Joinvile, Brazil) were inserted to monitor internal temperature during both freezing and, primarily, thawing.

### 2.2. Ultrasound-Assisted Thawing

Chicken breast samples were thawed in an ultrasonic (US) bath (Elma Schmidbauer GmbH, model TI-H-10 MF2, Singen, Germany) until reaching an internal temperature of 12 °C. Six individually packaged cylindrical samples were used per treatment, which varied according to frequency (25 or 130 kHz), amplitude (60% or 100%), and operation mode (normal, degas, or sweep), with the bath water maintained at 12 °C (Table 1). A total of 16 treatments were conducted, each using six individually packaged cylindrical samples. The entire experiment was repeated three times on different days, and all analyses were performed in sextuplicate, resulting in 18 observations per treatment. To evaluate the influence of water agitation, a magnetic stirrer (Fisatom^®^, model 712, 500 rpm, São Paulo, Brazil) was coupled to the US bath in specific treatments. Refrigerated fresh chicken breast served as the control (treatment 1), traditional thawing without US or agitation corresponded to TWUS/NoAgit (treatment 2), and thawing without US but with agitation corresponded to TWUS/Agit (treatment 3). The remaining treatments combined different US parameters, and the exposure times were established according to the conditions described by Flores et al. [18].

Ultrasound power for the different treatments was determined according to Raso et al. [19], using the following formula:

Power (W)=(dT/dt)·Cp·M

where

- *Cp* = specific heat capacity of water (4.2 J g^−1^);
- *M* = mass of water in the ultrasonic bath (g);
- *dT*/*dt* = temperature increase in the bath water over 600 s.

### 2.3. Analyses Performed

All analyses described in this section were performed on the samples as described in Section 2.2, totaling 18 observations per treatment, obtained from three independent repetitions conducted on different days with analyses carried out in sextuplicate.

#### 2.3.1. Temperature and Thawing Time

**Figure 1:**
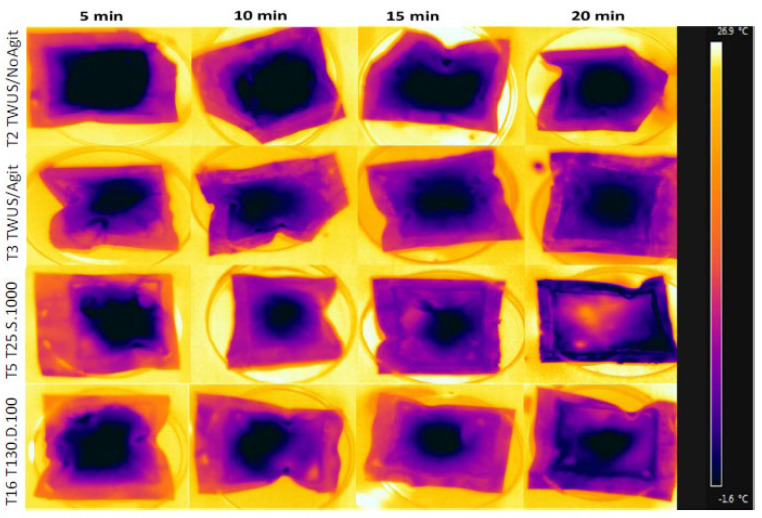
Thermographic images showing temperatures at different thawing times (5, 10, and 15 min) in chicken breasts from Treatment 2 (TWUS/NoAgit), Treatment 3 (TWUS/Agit), and with ultrasound in Treatments 5 (T25.S.100) and 13 (T130.100D).

Thawing temperature and time were evaluated by inserting thermocouples (Minipa MTK-15B, type K, Joinvile, Brazil) into the inner portion of the chicken breasts. These thermocouples were connected to a temperature measurement module (Maxim Semiconductor, Nanosheild model, San Jose, CA, USA). Samples were considered thawed when the internal temperature reached 12 °C. Additionally, packaged chicken breasts were imaged using an infrared thermal camera (FLIR^®^ Systems, Inc., model E60, Wilsonville, OR, USA) positioned at a distance of 10 cm. Images were taken immediately after removal from the freezer and after 5, 10, 15, and 20 min of thawing. These images were used to monitor thawing temperatures and to provide a visual representation of the thawing dynamics (Figure 1). Thermographic imaging was performed only for treatments 2 (TWUS/NoAgit), 3 (TWUS/Agit), 5 (T25.S.100), and 16 (T130.D.60), as they represent the conventional thawing controls and the ultrasound conditions with the best and worst thawing performance, thereby illustrating the range of responses without redundancy.

#### 2.3.2. Drip Loss

Drip loss from thawed chicken breasts was determined following the methodology of Zárate and Zaritzky [20], with adaptations for poultry samples. Immediately after thawing, samples of approximately 25 g were weighed and placed in polyethylene bags to prevent direct contact with the exudate. The packages were stored at 4 °C for 24 h, after which the samples were removed, gently blotted with filter paper to eliminate residual surface fluid, and reweighed. Drip loss was expressed as the percentage of weight lost during storage in relation to the initial weight.

#### 2.3.3. pH and Redox Potential (Eh)

pH and redox potential (Eh) were measured according to AOAC [21], using a pH meter (Digimed^®^, model DM 23 DC, São Paulo, Brazil) with a combined flow-type pH electrode (model DME-C1) and a combined platinum redox flow-type electrode (model DMR-CP1).

#### 2.3.4. Moisture Content

Moisture content was determined following the methodology of Marques et al. [22], using a microwave oven (Brastemp^®^, 30 L capacity, nominal power 820 W, actual power 662 W, 80% efficiency, São Paulo, Brazil). Tap water with a flow rate of 700 mL/min was used to absorb the radiation generated by the magnetron. Samples were weighed in polypropylene plastic cups and placed in the microwave oven for 12 min at maximum power. Moisture content was calculated using the following equation:

$$Moisture\left(\%\right)=100*(\frac{\left(Minitial-Mfinal\right)}{100})$$

where *Minitial* is the mass of the chicken breast cylinders before drying, and *Mfinal* is the mass after drying in the microwave oven.

#### 2.3.5. Water-Holding Capacity (WHC%)

Water-holding capacity was determined according to the methodology described by Kato et al. [23], with adaptations. Approximately 2.0 ± 0.10 g of sample were weighed, placed between two filter papers, and subjected to a 10.0 kg load for 5 min at room temperature. After compression, the samples were reweighed, and WHC was expressed as the percentage of retained water relative to the initial sample weight.

#### 2.3.6. Texture

Instrumental texture profile analysis (TPA) of chicken breasts was performed before freezing and after thawing using a texture analyzer (TA.XT.plus^®^, Stable Micro Systems, Surrey, UK), operated with Exponent software version 6.1.3.0. Samples were molded into cylindrical shapes (2 cm diameter × 1 cm thickness) and subjected to 50% compression with a cylindrical probe (P36) at a speed of 100.2 mm/min. Chewiness (N/m) and hardness (N) were evaluated.

#### 2.3.7. Myoglobin Redox States

The analysis of different myoglobin redox states (oxymyoglobin, deoxymyoglobin, metmyoglobin) and ferryl myoglobin radical followed the methodologies described by Tang et al. [24] and Wongwichian et al. [25].

#### 2.3.8. Color

Instrumental color analysis was performed using a spectrophotometer (Konica Minolta^®^, model CM-700, Tokyo, Japan) with illuminant A, including reflectance, 10° observer angle, and an average of two readings per sample, with a 3 mm SAV measurement area. L*, a*, and b* values were recorded. The overall color difference (∆E) was calculated for all treatments relative to the raw material (treatment 1), following AMSA [26]:

$$\Delta E=\surd ({\left(L*-L0\;*\right)}^{2}+{\left(a*-a0\;*\right)}^{2}+{\left(b*-b0\;*\right)}^{2})$$

where L*, a*, and b* represent lightness, redness, and yellowness of the treated samples, and L_0_, *a*_0_, and b*_0_ are the values of the untreated sample.

∆E values were categorized as: not perceptible (0–0.5), slightly perceptible (0.5–1.5), perceptible (1.5–3.0), clearly visible (3.0–6.0), and significantly different (6.0–12.0) [27].

#### 2.3.9. Total and Free Sulfhydryl Groups

Total and free sulfhydryl groups were determined according to the methodologies of Yin et al. [28], using Ellman’s reagent (DTNB). Absorbance was measured at 412 nm to calculate total sulfhydryl content, adopting the extinction coefficient of 13,600 M^−1^·cm^−1^. To determine reactive sulfhydryl groups, reaction mixtures were incubated without urea at 4 °C for 1 h. Results were expressed as µmol/g of protein.

#### 2.3.10. Statistical Analyses

Statistical analyses were performed using IBM SPSS Statistics software (SPSS, version 21, 2012). A generalized linear model (GLM) was used, with treatments considered as fixed effects and replicates as random effects. The random factor did not significantly contribute to the variability of the results (*p* > 0.05), and therefore was not retained in the final interpretation. Possible interactions between treatments and replicates were also tested but were not significant. Thus, only the main effects of treatments are reported. When significant differences were detected, analysis of variance (ANOVA) followed by Tukey’s test was applied at significance levels of *p* < 0.05. Results are presented as means ± standard deviation (*n* = 18).

## 3. Results and Discussion

### 3.1. Thawing Time of Chicken Breasts

All chicken breasts thawed under ultrasound (US) conditions exhibited significantly shorter thawing times (*p* < 0.05) compared with samples thawed without ultrasound. Using the conventional method (treatments 2: TWUS/NoAgit and 3: TWUS/Agit), thawing times were 50 and 39 min, respectively (Table 2). In contrast, sonicated samples showed markedly reduced times, particularly treatments 5 (T25.S.100, 18 min) and 6 (T25.S.100/Agit, 17 min), which used sweep mode at 25 kHz and 100% amplitude. At this frequency, the lower wavelength and higher intensity, combined with a high amplitude, enhance cavitation activity and release greater amounts of heat [29]. Consequently, thawing was reduced by 64% and 66% in treatments 5 and 6, respectively, compared with treatment 2 (Table 2).

**Figure 2:**
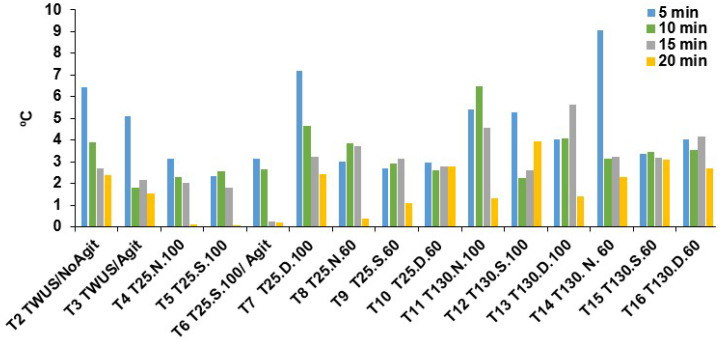
Temperature difference (°C) between the external part and the internal part of chicken breast cylinders, determined by sample thermography and the traditional process using thermocouples at thawing times of 5, 10, 15, and 20 min. Frequencies: 25 and 130 kHz; S: Sweep mode; D: Degas mode; N: Normal mode; 100 and 60 refer to ultrasound amplitudes.

Thermographic images taken at 5, 10, 15, and 20 min provided further insight into thawing dynamics (Figure 1). For treatment 5 (T25.S.100), external (T°e) and internal (T°i) temperatures were 7.0 and 3.88 °C at 5 min, 10.78 and 8.25 °C at 10 min, 13.70 and 11.68 °C at 15 min, and 14.88 and 15.45 °C at 20 min. These data indicate that thawing was essentially complete between 10 and 18 min (Figure 2). In contrast, for treatment 2 (TWUS/NoAgit), T°e and T°i reached only 9.15 and 6.78 °C after 20 min, and in treatment 3 (TWUS/Agit), 10.85 and 9.33 °C, indicating that thawing extended beyond 20 min.

The faster thawing observed in sonicated samples can be attributed to cavitation energy being absorbed preferentially in the internal regions (T°i) of the breast rather than at the surface (T°e). This phenomenon accelerates thawing, unlike conventional conduction-based thawing, in which heat is transferred gradually from the outer thawed layers to the frozen core [30].

Figure 2 further illustrates the influence of US frequency, amplitude, and operating mode by showing differences between T°e and T°i during thawing. For example, in treatment 12 (T130.S.100), differences were 5.28 °C, 2.23 °C, 2.58 °C, and 3.93 °C at 5, 10, 15, and 20 min, corresponding to a total thawing time of 24 min. In treatment 13 (T130.D.100), the respective differences were 4.05, 4.08, 5.63, and 1.40 °C, with thawing completed in 26 min. Similarly, in treatment 7 (T25.D.100), differences of 7.80, 4.65, 3.20, and 2.40 °C resulted in a thawing time of 26 min.

The operating mode can explain differences between treatments using the same frequency and amplitude. In normal mode, cavitation occurs at a constant frequency, optimizing liquid flow in the bath. In degas mode, cavitation bubbles are periodically displaced to the surface, while in sweep mode, the cavitation band is narrower, more uniform, and more intense [31]. These characteristics explain why sweep mode at 25 kHz and 100% amplitude (treatments 5 and 6) yielded the shortest thawing times (Table 2), with higher internal temperatures during thawing (Figure 2).

Mechanical agitation also affected thawing. Treatment 3 (TWUS/Agit) reduced thawing time by 23% compared with treatment 2 (TWUS/NoAgit), indicating that agitation of bath water improved heat transfer during conventional thawing. However, agitation had little effect under US. The difference between treatment 6 (T25.S.100/Agit) and treatment 5 (T25.S.100) was only 4.27%, demonstrating that thawing was primarily driven by US-induced cavitation rather than mechanical mixing.

### 3.2. Drip Loss (Exudation)

Treatment 10 (T25.D.60, 3.56%) showed the lowest drip loss, though not significantly different (*p* > 0.05) from treatments 4 (T25.N.100, 3.91%) and 6 (T25.S.100/Agit, 4.17%). The highest exudation values occurred in treatments 14 (T130.N.60, 9.05%) and 16 (T130.D.60, 8.72%), which differed significantly (*p* < 0.05) from all other treatments (Table 2).

These differences are linked to the frequency, amplitude, and operating mode applied. Frequencies of 130 kHz at 60% amplitude correspond to low-intensity conditions, which, combined with degas mode, act predominantly at the surface. This superficial action prolongs thawing, leading to limited modification of surface proteins and greater water release [32]. Conversely, 25 kHz in sweep or normal modes at 100% amplitude promoted faster thawing and lower drip loss. Under these conditions, sonication occurs with higher intensity, inducing structural rearrangements in both surface and internal proteins, exposing more hydrophilic groups that bind water, thereby reducing exudation [33].

### 3.3. Moisture Content

Only treatment 7 (T25.D.100, 71.23%) exhibited significantly lower moisture content (*p* < 0.05) compared with raw chicken breast (treatment 1, 72.67%), representing a reduction of 1.44%. Treatments 7 (71.23%), 12 (T130.S.100, 71.68%), 13 (T130.D.100, 71.78%), and 14 (T130N60, 71.64%) also showed lower values (*p* < 0.05) than treatment 2 (TWUS/NoAgit, 72.95%). However, treatments 5 (T25.S.100) and 6 (T25.S.100/Agit), which produced the shortest thawing times, did not differ (*p* > 0.05) from treatments 1, 2, and 3 in moisture content (Table 2).

These results reflect the physical effects induced by US, particularly the conversion of acoustic energy into heat. Heat transfer intensity varies with frequency, amplitude, and mode, leading to different thawing rates. In treatments 12 (T130.S.100) and 13 (T130.D.100), superficial overheating may have occurred due to limited cavitation penetration, impairing meat quality by promoting surface dehydration. When US is applied efficiently, thawing rates improve without causing overheating or excessive dehydration, thereby preserving quality [34].

### 3.4. Water-Holding Capacity (WHC)

In conventional thawing, WHC values were 17.03% (treatment 2, TWUS/NoAgit) and 17.70% (treatment 3, TWUS/Agit), which differed from all other treatments except treatments 4 (T25.N.100, 17.88%) and 8 (T25.N.60, 16.82%) (Table 2). WHC is one of the most critical attributes of meat quality, closely associated with juiciness, texture, and flavor [35].

Treatments 5 (T25.S.100, 23.14%) and 6 (T25.S.100/Agit, 21.58%) exhibited the highest WHC values (*p* < 0.05), significantly exceeding all other sonicated treatments. Slow freezing typically generates large ice crystals that damage muscle fibers, reducing water retention [36]. Therefore, the high WHC values in treatments 5 and 6 indicate that these samples would retain water during cooking and could be suitable for producing emulsion-type meat products. The improvement is associated with 25 kHz frequency, 100% amplitude, and sweep mode, which induced structural changes in both surface and internal proteins, exposing hydrophilic groups and enhancing water binding [13,37].

By contrast, treatments 7 (T25.D.100, 13.45%), 9 (T25.S.60, 13.68%), 10 (T25.D.60, 12.65%), 14 (T130.N.60, 9.69%), 15 (T130.S.60, 11.30%), and 16 (T130.D.60, 12.95%) showed significantly lower WHC values (*p* < 0.05) than treatments 2 and 3. The combination of frequency, amplitude, mode, and exposure time determines sonomechanical effects, including shock waves, high pressure, microjets, and turbulence. These effects modify protein structures, initially reducing molecular size and exposing hydrophilic groups, which enhances solubility and WHC [38].

### 3.5. pH and Redox Potential (Eh)

The raw chicken breast (treatment 1) had a pH of 5.89, which did not differ (*p* > 0.05) from treatments thawed without ultrasound: 2 (TWUS/NoAgit, 5.98) and 3 (TWUS/Agit, 5.98) (Table 2). Freezing and thawing promote denaturation of buffering proteins and release of H+ ions, typically leading to pH reduction [39]. However, treatment 4 (T25.N.100, 6.33) showed the highest pH value, which did not differ (*p* > 0.05) from treatments 7 (T25.D.100, 6.19), 12 (T130.S.100, 6.17), and 14 (T130.N.60, 6.17), but was significantly higher than all other treatments, including the raw sample. This suggests that under these US conditions, buffering proteins may not have undergone substantial denaturation [39].

Redox potential (Eh) in the raw breast was 169 mV, significantly higher (*p* < 0.05) than in treatments 3 (TWUS/Agit, 126 mV), 4 (T25.N.100, 127 mV), 9 (T25.S.60, 146 mV), 10 (T25.D.60, 128.3 mV), 11 (T130.N.100, 138.7 mV), 15 (T130.S.60, 145.3 mV), and 16 (T130.D.60, 129.2 mV) (Table 2). In contrast, treatments 2, 5, 6, 7, 8, 12, 13, and 14 did not differ significantly (*p* > 0.05) from the raw breast. Reduced Eh values in certain treatments may reflect enhanced interaction between antioxidant compounds and oxidized molecules facilitated by drip loss during thawing. Redox potential is influenced by oxygen availability, temperature, concentration of oxidizable/reducible substrates, and processing conditions [40].

### 3.6. Texture: Chewiness and Hardness

The chewiness of raw breast (treatment 1) was 44.29 N/m. Freezing and thawing increased chewiness across all treatments (Table 3). Conventional thawing (treatments 2 and 3) produced increases of 26.26% and 19.24% (*p* < 0.05) compared with treatment 1. Sonication at 25 kHz and 100% amplitude (treatments 4–6) increased chewiness by 21.72–41.52%, while at 25 kHz and 60% amplitude (treatments 7–10), the increase ranged from 13.72% to 38.63%. At 130 kHz and 100% amplitude (treatments 11–13), chewiness increased most sharply, by 40.73–48.34% (*p* < 0.05). At 130 kHz and 60% amplitude (treatments 14–16), increases ranged from 15.15% to 22.27%, similar to conventional thawing.

Hardness also increased in all treatments compared with the raw sample (48.27 N). In conventional thawing (treatments 2 and 3), hardness rose by 10.25–11.72% (*p* < 0.05). At 25 kHz and 100% amplitude, increases ranged from 6.57% to 19.58%; at 25 kHz and 60% amplitude, 12.99–20.07%. At 130 kHz and 100% amplitude, hardness increased by 27.76–34.43%, while at 60% amplitude the increase was 8.27–18.93% (Table 3).

The large extracellular ice crystals formed during slow freezing disrupted muscle fibers, reducing WHC (Table 2). This loss of water-binding capacity was inversely associated with increased chewiness and hardness (Table 3) [36]. For US to improve texture, energy input must be within an optimal range; too low or too high can have adverse effects, explaining the variability observed across treatments [41].

The high-intensity combinations (25 kHz, 100% and 60% amplitude) promoted rapid thawing (particularly in sweep mode) and reduced exudation (Table 2). Disulfide cross-linking contributed to increased hardness (Table 3). Conversely, at 130 kHz and both amplitudes, cavitation was more superficial, with limited water retention (Table 2), reflected in the lower hardness and chewiness values [5,33].

### 3.7. Myoglobin Redox States

Meat color is largely determined by the redox state of myoglobin, which is influenced by oxidative changes during storage and thawing [36]. Therefore, the levels of oxymyoglobin (OxyMb), deoxymyoglobin (DeoxyMb), metmyoglobin (MetMb), and ferryl myoglobin (FerrylMb) were analyzed in thawed samples.

#### 3.7.1. Oxymyoglobin (OxyMb)

OxyMb contains oxygen bound to the heme iron, imparting a bright red color preferred by consumers. In chicken, OxyMb is more abundant in thigh and drumstick muscles but is also present in lateral breast regions [42]. Treatments 1 (15.54%), 8 (15.50%), 10 (14.76%), 11 (14.87%), and 16 (15.57%) had the highest OxyMb values, which did not differ (*p* > 0.05) but were significantly higher (*p* < 0.05) than other treatments (Table 4). In contrast, treatments 2 (13.43%) and 3 (12.59%) showed reduced OxyMb after thawing, attributed to longer thawing times (50 and 39 min) and structural damage from large ice crystals, which facilitated OxyMb loss with drip water [36]. Treatment 13 (11.57%) also had significantly lower OxyMb (*p* < 0.05). At 130 kHz and degas mode, cavitation near the surface favored H_2_O_2_ formation, which oxidized OxyMb to MetMb [43].

#### 3.7.2. Deoxymyoglobin (DeoxyMb)

Treatments 4 (24.68%), 5 (25.40%), 6 (25.38%), 7 (25.00%), 8 (25.61%), 9 (25.53%), 10 (25.59%), 15 (25.35%), and 16 (25.41%) did not differ from the raw sample (25.12%). In contrast, treatments 2, 3, 11, 12, and 14 had significantly higher DeoxyMb (*p* < 0.05), suggesting lower oxygen availability, favoring conversion to MetMb [44].

#### 3.7.3. Metmyoglobin (MetMb)

Slow-frozen beef and pork (longissimus thoracis) also show increased MetMb upon thawing [45]. Treatment 13 (T130.D.100, 64.63%) had the highest MetMb (*p* < 0.05), consistent with conditions that favored oxidation of heme iron. Degas mode removes dissolved oxygen, promoting DeoxyMb oxidation [31], while US may inhibit glutathione oxidase activity, which normally reduces MetMb back to DeoxyMb [46].

#### 3.7.4. Ferryl Myoglobin (FerrylMb)

Pro-oxidants destabilize myoglobin, converting MetMb to FerrylMb. During auto-oxidation, superoxide (O_2_^−^) is generated, oxidizing Mb to the Ferryl cation radical, which propagates oxidation of proteins and lipids [47]. Slow freezing and thawing enhance contact between pro-oxidants, Mb, lipids, and proteins [39]. This explains the higher FerrylMb in treatments 2 (7.56 μM/kg) and 3 (17.04 μM/kg) compared with the raw breast (3.67 μM/kg). Treatment 3, with agitation, showed the highest FerrylMb, likely due to enhanced pro-oxidant release.

US treatments also produced ROS such as OH•, H•, O^−^, and OOH^−^, which can combine to form H_2_O_2_, a strong pro-oxidant [43]. The highest FerrylMb was in treatment 8 (18.57 μM/kg, *p* < 0.05), probably due to H_2_O_2_ formation in addition to freeze–thaw effects. In contrast, treatment 11 (2.92 μM/kg) had the lowest FerrylMb among sonicated samples, not differing from raw meat, suggesting minimal US effect. Treatments 5 (9.89 μM/kg) and 6 (10.75 μM/kg), which thawed fastest, also showed relatively low FerrylMb formation, likely because reduced exposure time limited ROS accumulation. In other US treatments (4, 7, 12, 13, 14, 15), FerrylMb ranged from 9.71 to 14.60 μM/kg, higher than in conventional thawing (treatment 2).

### 3.8. Instrumental Color

Because color is directly linked to myoglobin oxidation, L* (lightness) and overall color difference (ΔE) were analyzed relative to raw breast (treatment 1).

#### 3.8.1. L* (Lightness)

L* values are associated with free water content [30]. The raw breast (treatment 1) had L* of 54.34. Treatments 2 (53.20), 3 (53.56), 6 (54.03), 7 (53.77), 9 (54.75), and 11 (53.56) did not differ (*p* > 0.05) from the raw sample. In contrast, treatments 4 (51.42), 8 (51.68), 14 (50.80), and 15 (51.62) showed significantly lower values. Lower lightness is consistent with water loss during thawing, since large extracellular ice crystals disrupt protein structures, exacerbating water release [48]. Depending on US conditions, cavitation may or may not expose hydrophilic protein side chains. In treatments where exposure was limited, less free water was retained, reducing L*.

#### 3.8.2. Overall Color Difference (ΔE)

In conventional thawing (treatments 2 and 3), ΔE values were 3.91 and 2.85, respectively (Table 5). These values indicate well-visible (3.0–6.0) and perceptible (1.5–3.0) changes [27], reflecting the impact of slow freezing and long thawing times on cell and myofibrillar damage. Structural disruption facilitated Mb leakage with drip water, negatively affecting color [36].

Among US treatments, treatment 9 (T25.S.60) showed the lowest ΔE (2.74, perceptible). The other 13 US treatments showed well-visible differences (3.0–6.0). Depending on frequency and amplitude, US induced different levels of muscle structural changes, affecting water and Mb migration and thereby color and texture [37]. For example, treatments 8 (4.79), 12 (4.24), 14 (4.81), and 16 (5.21) produced particularly visible changes. These conditions likely caused superficial overheating due to low cavitation penetration, leading to dehydration (drip loss: 5.53, 6.72, 9.05, and 8.72%, respectively; Table 2) and Mb loss, which accentuated color differences [13].

### 3.9. Total and Free Sulfhydryl Groups

#### 3.9.1. Total Sulfhydryl Groups

All thawed samples exhibited significantly lower total sulfhydryl content (*p* < 0.05) compared with the raw breast (50.81 μmol/g protein). Reductions ranged from 9.00% (treatment 14, T130.N.60) to 37.36% (treatment 13, T130.D.100) (Table 6). Conventional thawing (treatments 2 and 3) resulted in decreases of 32.47% and 34.92%, respectively. Treatments 10 (37.12%) and 13 (37.36%) showed the most severe losses, not differing from treatment 3 but greater than most US treatments. Treatments 5 (30.72%) and 6 (27.66%) showed smaller decreases.

The formation of large extracellular ice crystals during slow freezing increases osmotic pressure, denatures proteins, and exposes sulfhydryl groups, which then undergo oxidation and form disulfide bonds [49]. During thawing, water from melted ice further facilitates oxidative interactions with proteins and lipids. Thus, thawing time and drip loss (Table 2) are key contributors to protein oxidation [36].

In sonicated samples, US effects overlapped with ice-crystal damage. Different frequencies, amplitudes, and operating modes produced varying intensities of cavitation, generating ROS such as OH• and H•. These radicals can oxidize sulfhydryls to sulfonic acids (R–SO_2_–OH) or form H_2_O_2_, which promotes further radical formation and disulfide cross-linking [50].

#### 3.9.2. Free Sulfhydryl Groups

According to Ying Lv et al. [49], lower sulfhydryl content indicates more extensive protein oxidation and reduced meat quality. Raw breast contained 30.82 μmol/g protein, not significantly different (*p* > 0.05) from treatment 10 (30.25). In contrast, conventional thawing (treatments 2 and 3) reduced free sulfhydryls by 46.85% and 43.41%, respectively. These losses reflect the combined effect of large ice crystals and prolonged thawing times.

All sonicated samples retained higher free sulfhydryls than treatments 2 and 3. The lowest values among US groups were observed in treatments 4 (24.21 μmol/g protein), 5 (24.69 μmol/g protein), 7 (23.27 μmol/g protein), and 13 (23.29 μmol/g protein). Treatments 5 and 6, which thawed fastest, showed decreases of 19.89% and 16.68%, respectively, smaller than those in treatments 2 (16.38%) and 3 (17.44%). This indicates that US not only accelerated thawing but also limited excessive oxidation.

US modifies protein quaternary and tertiary structures, exposing hidden sulfhydryl groups [49,50]. In treatments 5 and 6, rapid thawing reduced the exposure time to ROS while increasing the number of accessible sulfhydryls, thereby mitigating oxidative losses. In contrast, conventional thawing allowed prolonged contact between pro-oxidants and proteins, causing severe depletion.

Although treatment 10 (T25D60) exhibited a free thiol content comparable to raw meat, its water-holding capacity was lower. This apparent discrepancy can be explained by the fact that free thiol groups primarily reflect oxidative modifications, whereas water-holding capacity is strongly influenced by the structural integrity of the myofibrillar matrix. In this treatment, the lower ultrasound amplitude and degas mode may have reduced oxidative damage, preserving sulfhydryl groups, but at the same time promoted structural disruption through cavitation and mechanical stress, leading to higher exudation and lower water retention. Therefore, the results suggest that oxidation status and WHC are not directly correlated and can be differentially affected by ultrasound parameters.

## 4. Conclusions

In this study, ultrasound-assisted thawing was evaluated as an innovative strategy to improve the quality of slowly frozen chicken breast. All ultrasound treatments significantly reduced thawing time compared with conventional methods. However, the quality responses varied according to the applied parameters, including frequency, amplitude, and operating mode.

Among the evaluated conditions, treatments at 130 kHz and 60% amplitude (especially in degas mode) resulted in lower water-holding capacity, higher drip loss, increased protein oxidation, and more pronounced color changes, thereby compromising meat quality. In contrast, the optimized condition (25 kHz, 100% amplitude, sweep mode) provided the shortest thawing time, highest water-holding capacity, minimal exudation, reduced protein oxidation, and limited color alteration.

Overall, these findings highlight the potential of ultrasound, when properly optimized, as an effective technology to accelerate thawing while preserving the physicochemical quality of poultry meat. This study provides new insights into the optimization of ultrasound-assisted thawing and supports its future application and scaling in the poultry industry.

## Figures and Tables

**Table 1:** Frequencies, amplitudes, operating modes, and power in the ultrasound treatments applied to slow-frozen chicken breasts (*M. pectoralis major*).

Treatments		Frequency (kHz)	Amplitude (%)	Operating Modes	Power (W)
Number	Code				
1	Raw material	n.a	n.a	n.a	n.a
2	TWUS/NoAgit.	n.a	n.a	n.a	n.a
3	TWUS/Agit.	n.a	n.a	n.a	n.a
4	T25N100	25	100	Normal	202
5	T25S100	25	100	Sweep	218
6	T25S100/Agit.	25	100	Sweep	218
7	T25D100	25	100	Degas	168
8	T25N60	25	60	Normal	120
9	T25S60	25	60	Sweep	123
10	T25D60	25	60	Degas	112
11	T130N100	130	100	Normal	120
12	T130S100	130	100	Sweep	120
13	T130D100	130	100	Degas	108
14	T130N60	130	60	Normal	112
15	T130S60	130	60	Sweep	112
16	T130D60	130	60	Degas	95

Raw material: unfrozen chicken breast. Treatment 2 (TWUS/NoAgit): thawing in water without ultrasound and without stirring. Treatment 3 (TWUS/Agit): thawing in water without ultrasound but with stirring. n.a.: not applicable. Agit.: with stirring. Frequencies: 25 and 130 kHz. Amplitudes: 60% and 100%. Ultrasound bath operating modes: normal (N), sweep (S), and degas (D).

**Table 2:** Thawing time, drip loss, moisture content, water-holding capacity (WHC), pH, and redox potential (Eh) in ultrasound-thawed chicken breasts.

Treatments		Time (min)	Drip Loss (%)	Moisture (%)	WHC (%)	pH	Eh (mV)
Number	Code						
1	Raw material	n.a	n.a	72.67 ± 0.62 ^ab^	14.81 ± 0.42 ^gh^	5.89 ± 0.22 ^f^	169.00 ± 3.69 ^a^
2	TWUS/NoAgit.	50 ± 0.61 ^a^	4.65 ± 0.27 ^f^	72.95 ± 0.88 ^a^	17.03 ± 0.38 ^e^	5.98 ± 0.02 ^def^	165.83 ± 5.88 ^ab^
3	TWUS/Agit.	39 ± 0.58 ^b^	4.34 ± 0.26 ^fg^	72.36 ± 0.49 ^ab^	17.70 ± 0.84 ^e^	5.98 ± 0.06 ^def^	126.00 ± 4.15 ^d^
4	T25N100	25 ± 0.58 ^de^	3.91 ± 0.22 ^gh^	72.16 ± 0.34 ^ab^	17.88 ± 0.86 ^de^	6.33 ± 0.07 ^a^	127.00 ± 4.05 ^d^
5	T25S100	18 ± 0.50 ^g^	4.23 ± 0.22 ^fg^	72.43 ± 0.49 ^ab^	23.14 ± 0.93 ^a^	6.00 ± 0.09 ^cdef^	162.50 ± 4.89 ^ab^
6	T25S100/Agit.	17 ± 0.68 ^g^	4.17 ± 0.20 ^fgh^	72.05 ± 0.50 ^abc^	21.58 ± 0.63 ^b^	6.04 ± 0.04 ^bcdef^	162.16 ± 7.28 ^ab^
7	T25D100	26 ± 0.59 ^cde^	5.51 ± 0.22 ^de^	71.23 ± 0.30 ^c^	13.45 ± 0.76 ^hi^	6.19 ± 0.06 ^ab^	170.00 ± 6.54 ^a^
8	T25N60	27 ± 0.55 ^c^	5.53 ± 0.27 ^de^	72.29 ± 0.59 ^abc^	16.82 ± 0.93 ^ef^	6.12 ± 0.06 ^bcd^	164.00 ± 6.23 ^ab^
9	T25S60	23 ± 0.58 ^f^	5.46 ± 0.49 ^e^	72.73 ± 0.57 ^ab^	13.68 ± 0.40 ^hi^	6.12 ± 0.07 ^bcd^	146.00 ± 2.28 ^c^
10	T25D60	25 ± 0.58 ^cde^	3.56 ± 0.21 ^h^	72.10 ± 0.43 ^abc^	12.65 ± 0.76 ^ij^	6.03 ± 0.09 ^bcdef^	128.33 ± 4.18 ^d^
11	T130N100	26 ± 0.53 ^de^	5.56 ± 0.18 ^de^	71.91 ± 0.64 ^abc^	15.58 ± 0.81 ^fg^	5.95 ± 0.05 ^ef^	138.67 ± 3.14 ^c^
12	T130S100	24 ± 0.61 ^ef^	6.10 ± 0.87 ^de^	71.68 ± 0.47 ^bc^	19.34 ± 0.28 ^c^	6.17 ± 0.04 ^ab^	161.83 ± 5.64 ^ab^
13	T130D100	26 ± 0.55 ^cd^	6.72 ± 0.24 ^c^	71.78 ± 0.51 ^bc^	19.26 ± 0.53 ^cd^	6.08 ± 0.07 ^bcde^	165.83 ± 1.33 ^ab^
14	T130N60	25 ± 0.55 ^de^	9.05 ± 0.51 ^a^	71.64 ± 0.43 ^bc^	9.69 ± 0.50 ^k^	6.17 ± 0.06 ^ab^	158.33 ± 4.37 ^b^
15	T130S60	24 ± 0.64 ^f^	7.69 ± 0.36 ^b^	71.90 ± 0.15 ^abc^	11.30 ± 0.61 ^j^	6.06 ± 0.06 ^bcde^	145.33 ± 2.42 ^c^
16	T130D60	25 ± 0.50 ^de^	8.72 ± 0.49 ^a^	72.27 ± 0.87 ^abc^	12.95 ± 0.74 ^i^	6.03 ± 0.05 ^bcdef^	129.17 ± 3.43 ^d^

Values are expressed as mean ± standard deviation (SD). Different superscript letters within the same column indicate statistically significant differences between treatments according to Tukey’s test (*p* < 0.05). n.a.: not applicable. Treatments: See Table 1. Time (min): thawing time. WHC: water-holding capacity. Eh: redox potential (mV). Raw material: fresh meat prior to freezing.

**Table 3:** Texture values (chewiness and hardness) in chicken breasts thawed with and without ultrasound.

Treatments		Chewiness (N/m)	Hardness (N)
Number	Code		
1	Raw material	44.29 ± 1.31 ^g^	48.27 ± 1.2 ^g^
2	TWUS/NoAgit.	55.92 ± 2.76 ^cd^	53.22 ± 1.63 ^ef^
3	TWUS/Agit.	52.81 ± 2.51 ^cdef^	53.93 ± 1.1 ^def^
4	T25N100	54.63 ± 2.09 ^cde^	52.48 ± 2.06 ^ef^
5	T25S100	61.15 ± 3.06 ^b^	57.50 ± 1.2 ^cd^
6	T25S100/Agit.	62.68 ± 2.73 ^ab^	57.72 ± 1.01 ^bcd^
7	T25D100	53.91 ± 1.24 ^cdef^	51.49 ± 1.83 ^fg^
8	T25N60	52.76 ± 2.00 ^cdef^	55.11 ± 2.64 ^def^
9	T25S60	61.40 ± 1.89 ^b^	57.96 ± 2.54 ^bcd^
10	T25D60	50.39 ± 1.44 ^f^	54.54 ± 2.43 ^def^
11	T130N100	62.33 ± 1.03 ^ab^	64.89 ± 1.8 ^a^
12	T130S100	63.97 ± 2.46 ^ab^	61.67 ± 1.52 ^ab^
13	T130D100	65.70 ± 2.88 ^a^	60.79 ± 2.7 ^bc^
14	T130N60	56.37 ± 1.13 ^c^	57.41 ± 1.42 ^cd^
15	T130S60	51.00 ± 1.24 ^ef^	52.26 ± 2.59 ^efg^
16	T130D60	51.88 ± 1.72 ^def^	55.77 ± 2.73 ^de^

Values are expressed as mean ± standard deviation (SD). Different superscript letters within the same column indicate statistically significant differences between treatments according to Tukey’s test (*p* < 0.05). Treatments: See Table 1. Raw material: fresh meat prior to freezing.

**Table 4:** Values of deoxymyoglobin (DeoxyMb), oxymyoglobin (OxyMb), metmyoglobin (MetMb), and ferryl myoglobin (FerrylMb) in chicken breasts thawed with and without ultrasound.

Treatments		DeoxyMb (%)	OxyMb (%)	MetMb (%)	FerrylMb (µM/kg)
Number	Code				
1	Raw material	25.12 ± 0.49 ^de^	15.54 ± 0.58 ^a^	61.55 ± 0.80 ^bc^	3.67 ± 0.17 ^k^
2	TWUS/NoAgit.	26.84 ± 0.45 ^a^	13.43 ± 0.44 ^fgh^	59.31 ± 0.55 ^ef^	7.56 ± 0.83 ^ij^
3	TWUS/Agit.	26.03 ± 0.94 ^abc^	12.59 ± 0.06 ^hi^	60.69 ± 0.83 ^cde^	17.04 ± 0.78 ^b^
4	T25N100	24.68 ± 0.11 ^ef^	12.60 ± 0.34 ^h^	62.68 ± 0.95 ^b^	14.12 ± 0.73 ^cd^
5	T25S100	25.40 ± 0.25 ^def^	13.98 ± 0.64 ^cdef^	60.83 ± 0.47 ^cde^	9.89 ± 0.39 ^fg^
6	T25S100/Agit.	25.38 ± 0.13 ^cde^	13.65 ± 0.46 ^efgh^	61.25 ± 0.59 ^bcd^	10.75 ± 0.50 ^fg^
7	T25D100	25.00 ± 0.22 ^de^	13.82 ± 0.57 ^defg^	62.02 ± 0.99 ^bc^	9.71 ± 0.75 ^gh^
8	T25N60	25.61 ± 0.28 ^bcd^	15.50 ± 0.24 ^ab^	58.59 ± 0.91 ^f^	18.57 ± 0.64 ^a^
9	T25S60	25.53 ± 0.66 ^cd^	13.36 ± 0.59 ^fgh^	60.90 ± 0.87 ^cde^	6.56 ± 0.41 ^j^
10	T25D60	25.59 ± 0.28 ^bcd^	14.76 ± 0.67 ^abcd^	59.87 ± 0.96 ^def^	11.02 ± 0.54 ^f^
11	T130N100	26.04 ± 0.19 ^abc^	14.87 ± 0.50 ^abc^	59.26 ± 0.57 ^ef^	2.92 ± 0.55 ^k^
12	T130S100	26.39 ± 0.33 ^ab^	14.27 ± 0.64 ^cdef^	59.69 ± 0.85 ^def^	13.12 ± 0.64 ^de^
13	T130D100	24.12 ± 0.47 ^f^	11.57 ± 0.41 ^i^	64.63 ± 0.76 ^a^	12.64 ± 0.47 ^e^
14	T130N60	26.10 ± 0.08 ^abc^	14.49 ± 0.45 ^bcde^	59.58 ± 0.50 ^ef^	14.60 ± 0.58 ^c^
15	T130S60	25.35 ± 0.31 ^cde^	12.92 ± 0.58 ^gh^	62.15 ± 0.95 ^bc^	14.18 ± 0.76 ^cd^
16	T130D60	25.41 ± 0.30 ^cde^	15.57 ± 0.38 ^a^	58.84 ± 0.94 ^f^	8.52 ± 0.14 ^hi^

Values are expressed as mean ± standard deviation (SD). Different superscript letters within the same column indicate statistically significant differences between treatments according to Tukey’s test (*p* < 0.05). Treatments: See Table 1. Raw material: fresh meat prior to freezing.

**Table 5:** Values of L* (lightness) and overall color difference (ΔE) in chicken breasts thawed with and without ultrasound.

Treatments		L*	ΔE
Number	Code		
1	Raw material	54.34 ± 1.03 ^bcd^	n.a.
2	TWUS/NoAgit.	53.20 ± 3.54 ^bcdef^	3.91 ± 2.14
3	TWUS/Agit.	55.04 ± 1.73 ^ab^	2.85 ± 0.92
4	T25N100	51.42 ± 2.69 ^fg^	3.61 ± 1.98
5	T25S100	52.26 ± 2.73 ^defg^	3.72 ± 1.43
6	T25S100/Agit.	54.03 ± 2.99 ^bcd^	3.92 ± 0.97
7	T25D100	53.77 ± 1.10 ^bcde^	3.25 ± 1.26
8	T25N60	51.68 ± 1.72 ^efg^	4.79 ± 0.82
9	T25S60	54.75 ± 0.52 ^bc^	2.74 ± 1.29
10	T25D60	52.70 ± 1.27 ^cedfg^	3.95 ± 1.49
11	T130N100	53.56 ± 0.85 ^bcdef^	3.44 ± 0.73
12	T130S100	51.44 ± 3.49 ^fg^	4.24 ± 2.21
13	T130D100	52.08 ± 1.77 ^defg^	3.33 ± 1.29
14	T130N60	50.80 ± 1.13 ^g^	4.81 ± 1.59
15	T130S60	51.62 ± 1.03 ^efg^	3.77 ± 2.15
16	T130D60	57.08 ± 1.70 ^a^	5.21 ± 2.38

Values are expressed as mean ± standard deviation (SD). Different superscript letters within the same column indicate statistically significant differences between treatments according to Tukey’s test (*p* < 0.05). ΔE: Overall color difference in relation to the raw material (unfrozen chicken breast). n.a.: not applicable. Treatments: See Table 1. Raw material: fresh meat prior to freezing.

**Table 6:** Values of total and free sulfhydryls in chicken breasts thawed with and without ultrasound.

Treatments		Sulfhydryls (µmol/g of Protein)	
Number	Code	Total	Free
1	Raw material	50.81 ± 0.92 ^a^	30.82 ± 0.66 ^a^
2	TWUS/NoAgit.	34.31 ± 0.57 ^fg^	16.38 ± 0.68 ^g^
3	TWUS/Agit.	33.07 ± 0.69 ^gh^	17.44 ± 0.68 ^g^
4	T25N100	43.43 ± 0.74 ^c^	24.21 ± 0.77 ^ef^
5	T25S100	35.20 ± 0.43 ^ef^	24.69 ± 0.35 ^def^
6	T25S100/Agit.	36.80 ± 0.53 ^e^	25.68 ± 0.99 ^de^
7	T25D100	34.31 ± 0.88 ^fg^	23.27 ± 0.15 ^f^
8	T25N60	34.47 ± 0.89 ^fg^	25.90 ± 0.65 ^cd^
9	T25S60	35.22 ± 0.7 ^ef^	27.37 ± 0.55 ^bc^
10	T25D60	31.95 ± 0.96 ^h^	30.25 ± 0.55 ^a^
11	T130N100	39.81 ± 0.9 ^d^	28.70 ± 0.72 ^b^
12	T130S100	43.66 ± 0.52 ^c^	25.46 ± 0.27 ^de^
13	T130D100	31.83 ± 0.6 ^h^	23.29 ± 0.16 ^f^
14	T130N60	46.24 ± 0.72 ^b^	27.27 ± 0.43 ^bc^
15	T130S60	36.74 ± 0.43 ^e^	25.05 ± 0.77 ^de^
16	T130D60	34.78 ± 0.85 ^fg^	25.50 ± 0.34 ^de^

Values are expressed as mean ± standard deviation (SD). Different superscript letters within the same column indicate statistically significant differences between treatments according to Tukey’s test (*p* < 0.05). Treatments: See Table 1. Raw material: fresh meat prior to freezing.

## Data Availability

The original contributions presented in this study are included in the article. Further inquiries can be directed to the corresponding author.
